# β-Glucans in particulate and solubilized forms elicit varied immunomodulatory and apoptosis effects in teleost macrophages in a dosedependent manner

**DOI:** 10.3389/fimmu.2023.1243358

**Published:** 2023-08-22

**Authors:** Zhelin Wu, Yanjian Yang, Jiadong Li, Peter Bossier, Xiayi Wei, Zheng Guo, Biao Han, Jianmin Ye

**Affiliations:** ^1^ Guangzhou Key Laboratory of Subtropical Biodiversity and Biomonitoring, Guangdong Provincial Engineering Technology Research Center for Environmentally-Friendly Aquaculture, School of Life Sciences, South China Normal University, Guangzhou, China; ^2^ Laboratory of Aquaculture & Artemia Reference Center, Department of Animal Sciences and Aquatic Ecology, Faculty of Bioscience Engineering, Ghent University, Gent, Belgium; ^3^ Guangdong Laboratory for Lingnan Modern Agriculture, Guangzhou, China

**Keywords:** β-Glucans, macrophage, immune responses, apoptosis, α-Ketoglutarate

## Abstract

β-Glucans are a group of heterogeneous glucose polymers that possess immunomodulatory activities. The complex nature of their structures, uncertainty regarding the doses, and variable immune effects pose a challenge to comprehensive understanding. In this study, we investigated the immune responses and apoptosis effects in Nile tilapia (*Oreochromis niloticus*) head kidney macrophages (MФ) upon exposure to two β-Glucans (Paramylon and Laminarin) at low and high doses. Our results demonstrate that Paramylon elicits more robust immune responses than Laminarin, albeit with a dose-limiting effect. We also observed that the high-dose Paramylon induces apoptosis, whereas no such effect was detected in Laminarin treatment. Mechanistically, high-dose Paramylon activates the intrinsic apoptosis pathway, with significantly up-regulation of intrinsic apoptosis-related genes and impaired mitochondrial function. On the other hand, Laminarin triggers metabolic reprogramming in MФ, resulting in the enrichment of the metabolite α-Ketoglutarate, which protects the MФ from apoptosis. Overall, our findings highlight the importance of identifying the optimal dose range for β-Glucans, based on sources or structures, to achieve maximal immunomodulatory effects. These results have important implications for the design and optimization of β-Glucans-based drugs or adjuvants in immunotherapies.

## 1 Introduction

β-Glucans, a class of polysaccharides, have gained attention for their diverse biological activities, particularly their immunomodulatory effects (1, 2). These properties have been attributed to their ability to interact with immune cells (3), either through direct binding to receptors or modulation of cytokine production, ultimately leading to the activation of innate and adaptive immune responses (4). However, the physicochemical properties and biological activities of β-Glucans can be significantly influenced by their structural variability, which is determined by their origin (5, 6). Additionally, the dose of β-Glucans is a variable factor that must be considered (7). Therefore, it is crucial to comprehend the structural diversity and optimal dose of β-Glucans to ensure their appropriate utilization in various applications.

β-Glucans are found in a variety of sources, including bacteria, fungi, yeast, seaweed and cereal grains, whose structures are primarily determined by the type of glycosidic linkage between glucose residues in the polymer backbone, as well as the degree and position of branching (8–10). Take Paramylon for an example, it has linear β- (1, 3) backbones devoid of branches and is extracted from *Euglena gracilis*, a new superfood source rich in natural β-Glucans (11). One distinguishing feature of Paramylon compared to other β-Glucans is its high yield, typically making up at least 90% of the dry weight of the host cell; hence, it is considered easier to extract a highly purified version of β-Glucan. Paramylon has been shown to exhibit immunological activity in various experimental models. Previous studies have reported that Paramylon activates human lymphocytes by increasing NF-κB trans-activation and up-regulating pro-inflammatory cytokines such as *TNF-α*, *IL-6* and *COX-2* (12). Similarly, it activates RAW264.7 macrophages in a dose-dependent manner via the MAPK signaling pathway (13). Meanwhile, the structure of β-Glucans extracted from the seaweed source varies slightly depending on the species. Some of them contain β- (1,3) straight chain residues, while others have β- (1, 3) main chains linked to varying amounts of β- (1,6) branches (14). Recent researches have reported that Laminarin (extracted from *Laminaria digitata*, a β- (1, 3)-D-glucan containing several β- (1,6) interchain junctions and branch points) activates reactive nitrogen species (RNS), reactive oxygen species (ROS) and hypochlorous acid in human neutrophils, thereby improving the killing of infectious pathogens (15). *In vitro* investigations have shown that Laminarin can stimulate ROS and cytokine production in neutrophils and monocytes of pigs; however, its stimulation of phagocytes is lower than that of other β-Glucans (16). Furthermore, when compared with 12 other β-Glucans, Laminarin has been found to induce the release of interleukins to a lesser extent in human whole blood (17). Nonetheless, soluble β-Glucans have been widely used in clinical applications because of their ease of *in vivo* delivery. In addition, Laminarin is more widely available and easier to extract than Paramylon, which makes it a more practical and cost-effective option for many industries. Therefore, it is essential to consider the structural differences, easiness of application, availability, and possible specific immunomodulatory effects of these two β-Glucans when assessing their potential health benefits.

When evaluating the benefits of any immunomodulator, it is important to acknowledge the dual nature of its effects, which also applies to β-Glucans. The optimal immunomodulatory effects are achieved with appropriate β-Glucan dosage, as higher dosages do not necessarily equate to better outcomes, and excessive doses can result in immunosuppression or apoptosis. For instance, a negative correlation between the concentration of β-Glucans and lobster granulocytes viability has been reported (18). Besides that, lower doses (1-2 g/kg) of β-Glucans administered to rainbow trout elicited upregulation of immune-related transcripts, enhancement of innate immune parameters, and enhanced resistance against *Aeromonas hydrophila* infection. Conversely, higher doses (5 g/kg) of β-Glucans induced a non-reactive physiological state in the fish (19). This observed outcome may be attributed to the potential phenomenon where an increased dose of β-Glucans led to an overwhelming influx of β-Glucans molecules into the immune cells, thereby inducing a state of excessive activation that ultimately impaired the immunological functions of the cells (20). Furthermore, *in vitro* experiments revealed that β-Glucans exerted a pronounced apoptotic effect on carp pronephric leucocytes, with a dose threshold of 500 μg/mL or higher (21).

Building upon the evolutionary conservation of the innate immune system and the fact that Paramylon and Laminarin can induce varying cellular immune effects in higher vertebrate, we sought to investigate the divergent effects of two distinct β-Glucans on head kidney MФ of Nile tilapia (*Oreochromis niloticus*). To this end, we stimulated the MФ by exogenously treatment of the β-Glucans at predetermined low and high doses. Considering the dosages utilized in previous cell experiments involving β-Glucans in teleost fish (**Supplemental Table 1**), we conducted preliminary experiments utilizing low (10 μg/mL), medium (50 μg/mL), and high (200 μg/mL) concentration gradients to evaluate the phagocytic capacity of MФ following β-Glucan stimulation (**Supplemental Figure 1**). Based on the observed discrepancies in results, we subsequently opted to use 10 μg/mL and 200 μg/mL as indicated low and high doses for the formal experiments. Our study revealed the distinct immune responses of Paramylon and Laminarin on tilapia MФ, and intriguingly, differential apoptotic effects were also observed between the two polysaccharides at the high-dose treatment. Furthermore, we applied multi-omics approaches and immunological techniques to elucidate the underlying mechanisms.

## 2 Materials and methods

### 2.1 Experimental animal and ethics statement

The Nile tilapia (*Oreochromis niloticus*) utilized in this experiment were procured from the Guangdong Tilapia Breeding Farm (Guangzhou, China) and housed at the Institute of Modern Aquatic Science and Engineering, South China Normal University. The fish were raised under controlled conditions in a temperature-regulated water recirculation system equipped with an automatic filtration system to ensure optimal health. The average weight of the experimental fish was maintained at 200 ± 20 g, and the culture temperature was kept at a constant 25 ± 2 °C. The culture environment was periodically monitored to maintain normal water oxygen solubility, nitrite and ammonia nitrogen levels. The experimental procedures followed the regulations of the South China Normal University Animal Care and Use Committee (SCNU-SLS-2021-009) and were carried out in accordance with the ARRIVE guidelines.

### 2.2 Reagents and preparation

Paramylon (CAS: 9051-97-2), a linear β- (1,3)-D-glucan without any branches, has a molecular weight of about 500,000 Da and is insoluble. Laminarin (CAS: 9008-22-4), a β- (1,3)-D-glucan containing several β- (1,6) interchain junctions and branch points, has a molecular weight up to 500Da and is soluble. Both commercial β-Glucans mentioned above were purchased from Sigma-Aldrich (USA). Enasidenib (AG-221; CAS: 1446502-11-9; Purity 99.97%) was purchased from Med Chem Express (USA). Dimethyl-α-Ketoglutarate (DMKG; CAS: 13192-04-6; Purity ≥ 95.5%) was purchased from Sigma-Aldrich (USA). Prior to use, β-glucans were dissolved in sterile PBS to a final concentration of 1 mg/mL, and insoluble Paramylon was mixed by repeated blowing with a pipettor.

### 2.3 Fluorescent labeling of bacteria

*Streptococcus agalactiae* (ZQ1901) was gifted by Prof. Jian from the Guangdong Key Laboratory of Pathogenic Biology and Epidemiology for Aquatic Economic Animals (Zhanjiang, China). For fluorescent labeling of bacteria (22), they were first heat inactivated at 60 °C for 40 min, followed by centrifugation and resuspension in sterile PBS. Subsequently, 2 mg/mL of FITC (Sigma-Aldrich, USA) was added to the bacterial suspension, which was then incubated at 160 rpm for 2 h at 30 °C under light-proof conditions. The completed incubations were washed more than four times with sterile PBS until the dye float was completely washed away. The labeled bacteria were then stored at -20 °C for use.

### 2.4 Isolation of Nile tilapia head kidney MФ

Macrophage isolation was based on a previously established and reliable method (23, 24). Briefly, the head kidney was aseptically removed and placed in a sterile 60 mm cell and tissue culture dish (JET BIOFIL, China) containing RPMI 1640 medium (Gibco, USA) supplemented with 1% penicillin/streptomycin (Gibco, USA). The tissue was gently homogenized with a sterile syringe and filtered through a 70 μm cell strainer (Corning, USA) to obtain a single cell suspension. Cell suspension with a 10 mL volume was layered over a density gradient stratification of 54%/31% Percoll (Sigma-Aldrich, USA) and centrifuged at 400 × *g* for 40 min at 4°C. Cells were collected from the 54%-31% partition level after centrifugation. Cells were washed with RPMI-1640, counted using 0.1% Trypan blue (Solarbio, China), and adjusted to a concentration of 1 × 10^6^ cells per well in RPMI-1640 complete medium containing 1% penicillin/streptomycin and 10% fetal bovine serum (FBS) (Gibco, USA) in a 96-well cell culture plate (Corning, USA). Non-adherent cells were removed after 24 h, and MФ was washed and cultured in complete medium for further experiments. All procedures were performed at 25°C in a sterile environment.

### 2.5 β-Glucans stimulation, RNA extraction, cDNA preparation and qRT-PCR

Quantitative stock solutions of Paramylon and Laminarin prepared as described in **2.2** were added directly to MФ cultured in 96-well plates to achieve the target stimulation doses (low dose: 10 μg/mL, and high dose: 200 μg/mL). Total RNA was isolated from indicated reagents treated/untreated 12 h MФ using RNA isolator (Vazyme, China) according to the manufacturer’s protocol, and then the purity and concentration of RNA were determined using an ultramicro UV-visible spectrophotometer (Nanodrop-2000) (Thermo, USA). cDNAs were synthesized by Hifair^®^ II 1st Strand cDNA Synthesis SuperMix for qPCR (gDNA digester plus) (YEASEN, China) and qRT-PCR was performed by CFX96 Touch (Bio-Rad, USA) with Hieff^®^ qPCR SYBR Green Master Mix (YEASEN, China). Relative mRNA levels were quantified using the 2^-ΔΔCt^ method and normalized to the internal control *β-actin*. All gene-specific primers are presented in **Table 1**.

**Table 1:** Primers used for qRT-PCR in this study.

Gene	Primers	Sequences (5′-3′)
*IL-1β*	qIL-1β F	GTTCACCAGCAGGGATGAGATT
	qIL-1β R	TGCGGTCTTCACTGCCTCC
*IL-6*	qIL-6 F	ACAGAGGAGGCGGAGATG
	qIL-6 R	GCAGTGCTTCGGGATAGAG
*IL-8*	qIL-8 F	GATAAGCAACAGAATCATTGTCAGC
	qIL-8 R	CCTCGCAGTGGGAGTTGG
*TNF-α*	qTNF-α F	GCTGAGGCTCCTGGACAAAA
	qTNF-α R	TCTGCCATTCCACTGAGGTCTT
*Bax*	qBax F	GGCAATAAAGCAGTGACGAGAG
	qBax R	ATTTCCATCCAGCTCGTCTCC
*Cyt-c*	qCyt-c F	TGTCCAGAAATGTTCCCAGTGC
	qCyt-c R	CCTTTGTTCTTATTGGCATCTGTG
*Apaf-1*	qApaf-1 F	CTGCTTCTCGCAGGATGGAA
	qApaf-1 R	GAGAAGGCACAGCAAAGCAC
*caspase-7*	qcaspase-7 F	TGTCATTTCATTTACTTCACCAGGC
	qcaspase-7 R	GCCTCCACCGGGATCTTATG
*β-actin*	qβ-actin F	CGAGAGGGAAATCGTGCGTGACA
	qβ-actin R	AGGAAGGAAGGCTGGAAGAGGGC

### 2.6 Detection of MФ phagocytosis and ROS levels

Three phagocytosis models including phagocytosis of *S. agalactiae*, 0.5 and 1.0 μm Fluoresbrite^®^ YG carboxylate microspheres (YG beads) (Polysciences Inc., USA) were established to evaluate the phagocytosis of MФ after 12h β-Glucans stimulation. As described in our previous studies (25, 26), the co-incubation of treated/untreated MФ with phagocytic objects was performed at 25°C under light-proof conditions for 4 h, at a ratio of cells to FITC-labeled bacteria or YG beads of 1:20. The number of cells in each sample was 1 × 10^6^. Following incubation, cells were washed three times with ice-cold sterile PBS to remove excess and adherent bacteria or YG beads on the cell surface. The level of phagocytosis was determined using flow cytometry (BD LSRFortessa™) (BD Biosciences, USA). The region of positivity was defined using control cells that were not subjected to any treatment, and the percentage of positive cells was indicative of the intensity of phagocytosis. Flow cytometry data were analyzed and visualized using FlowJo 10.8.1. ROS detection was also performed by flow cytometry using the ROS Assay Kit (Beyotime, China) according to the manufacturer’s instructions, and ROS levels were measured as mean fluorescence intensity (MFI).

### 2.7 Apoptosis assay

The apoptosis assay was conducted in accordance with the protocol provided by the FITC Annexin V Apoptosis Detection Kit I (BD Biosciences, USA). Specifically, MФ that had been treated/untreated with indicated reagents was collected in sterile 1.5 mL centrifuge tubes (Axygen, USA), centrifuged at 500 × *g* for 10 min at 4°C, and the supernatant was discarded. The pellet was then resuspended in 150 µL of Annexin V Binding Buffer, followed by sequential addition of FITC Annexin V and Propidium Iodide Staining Solution (PI) under dark conditions. After 15 min of incubation at 25°C, an additional 150 µL of Binding Buffer was added. The mixture was mixed by pipetting and transferred to a flow tube (BD Falcon, USA) for flow cytometry analysis. Blank cell samples without any treatment, as well as Annexin V FITC and PI mono-stained control cell samples, were also prepared to identify the positive areas of the data. The acquired data were analyzed and presented using FlowJo 10.8.1.

### 2.8 Transcriptome sequencing, untargeted metabolomics sequencing and bioinformatical analyses

Transcriptome sequencing was performed using RNA extracted from β-Glucans treated/untreated 12h MФ through DNBSEQ platform (BGI, China). Five groups of samples were used for transcriptome sequencing, including Control, Paramylon-low, Paramylon-high, Laminarin-low and Laminarin-high, with three parallel set in each group. Metabolomics sequencing was performed using high-resolution mass spectrometer (Q Exactive) (Thermo Fisher Scientific, USA) to collect data from both positive and negative ions to improve metabolite coverage, which was based on the LC-MS/MS technology. Metabolomics sequencing samples were divided into 3 groups: Control, Paramylon-low and Laminarin-low, with five parallel set in each group. The pretreatment time of β-Glucans treatment groups was 12h. KEGG pathway analysis was conducted using the Kyoto Encyclopedia of Genes and Genomes (KEGG) database to identify the enriched pathways of differentially expressed genes and metabolites.

### 2.9 Mitochondrial membrane potential and ROS measurements

To evaluate changes in mitochondrial membrane potential (MMP), we employed the Enhanced Mitochondrial Membrane Potential Assay Kit with JC-1 (Beyotime, China) and measured the relative ratio of red to green fluorescence (PE/FITC) using flow cytometry. Mitochondrial ROS (mtROS) was determined using the MitoSOX™ red mitochondrial superoxide indicator (Invitrogen, USA). MФ was loaded with MitoSOX diluted in HBSS (Gibco, USA) in darkness for 30 min at 25°C, and rinsed with warm HBSS before flow cytometry analysis. Additionally, we used Live Cell Staining Solution Hoechst 33342 (Beyotime, China) to stain the cells during MitoSOX loading, and visualized mtROS production in individual cells using a laser confocal scanning microscope (LSM710) (Carl Zeiss, Germany). Analysis of resulting fluorescence images were performed with ZEN 3.5.

### 2.10 Biochemical assay

Following the washing step with precooled PBS, intracellular ATP content was determined using the Enhanced ATP Assay Kit (Beyotime, China) as per the manufacturer’s instructions. Cellular α-Ketoglutarate (α-KG) was quantified using the α-KG Assay Kit (Sigma-Aldrich, USA). In brief, prepared equal volumes of samples and Reaction Mixes were added to a 96-well transparent microplate and read at a wavelength of 570 nm using a multifunctional microplate reader (EnSpire) (PerkinElmer, USA). The concentration of cellular α-KG was calculated using a linear regression equation for α-KG standards.

### 2.11 Statistical analysis

Data were presented as the means ± standard deviation (SD) of four independent experiments. Statistical significance was established by ANOVA with Tukey’s multiple comparison tests. Statistical analyses were performed in GraphPad Prism 9.3.1. The index *p* < 0.05 was considered statistically significant.

## 3 Results

### 3.1 Different structures and doses of β-Glucans lead distinct immune responses in MФ

**Figure 1:**
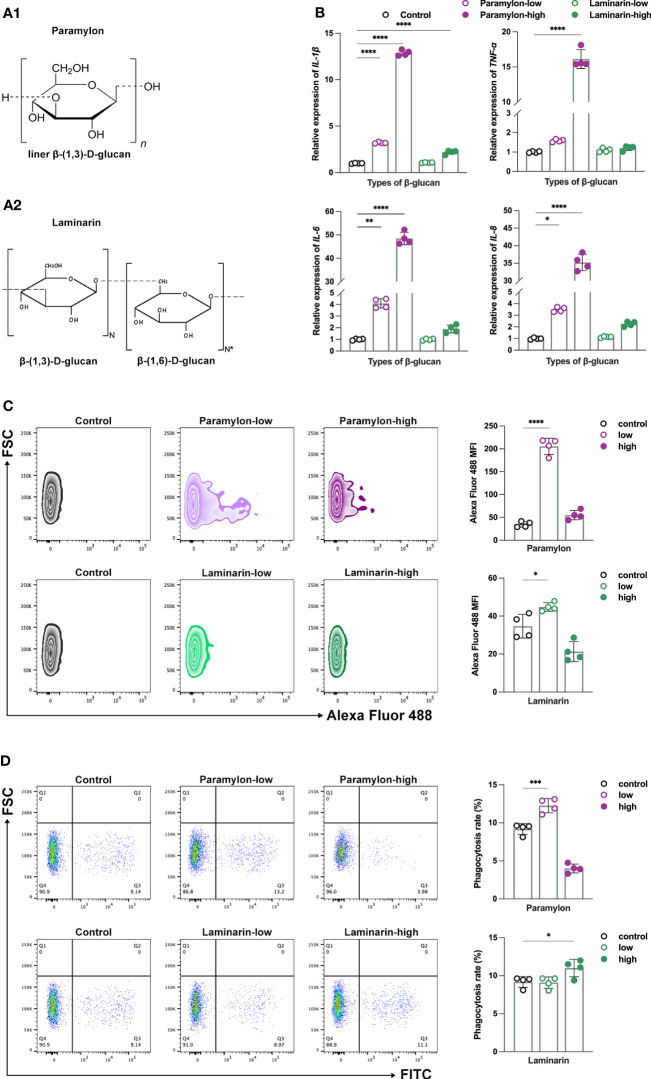
β-Glucans conferred differential immune responses. The chemical structures of Paramylon **(A1)** and Laminarin **(A2)**. Pictures originally published in (4, 27). **(B)** Relative expression patterns of pro-inflammatory cytokines *IL-1β*, *6*, *8* and *TNF-α* mRNA in both low (10 μg/mL) and high (200 μg/mL) -doses β-Glucans treated (12 h) MФ. **(C)** ROS levels and **(D)** phagocytosis performance of both low and high-doses β-Glucans treated (12 h) MФ against *S. agalactiae*. All statistical analyses of the data were expressed as means ± SD (n=4). Statistical significance was established by One-Way ANOVA. * *p* < 0.05, ** *p <*0.01, *** *p* < 0.001, **** *p* < 0.0001.

To investigate the potential differences in immune responses induced by variations in the structure and dose of β-Glucans, MФ was treated with Paramylon (**Figure 1A1**) and Laminarin (**Figure 1A2**), two distinct structural β-Glucans derived from *Euglena gracilis* and *Laminaria digitata*, respectively, at both low and high doses. We first tested the effect of exogenously added β-Glucans on the inflammatory response. The expressions of the measured pro-inflammatory cytokines, including *IL-1β*, *IL-6*, *IL-8* and *TNF-α*, were significantly upregulated with increasing doses of Paramylon (**Figure 1B**). Conversely, Laminarin demonstrated little change in cytokine expression at any dose tested (**Figure 1B**). Typically, reactive oxygen species (ROS) are produced in conjunction with a pro-inflammatory response (28). Consistent with the expression of cytokines, the ROS levels increased significantly in response to low-dose Paramylon treatment, but the rate of increase exhibited a diminishing trend at the high-dose treatment (**Figure 1C**). Furthermore, Laminarin exhibited a distinct behavior from Paramylon in terms of ROS production, even showing inhibition of that at high doses (**Figure 1C**).

As phagocytosis is a fundamental function of MФ (29), we proceeded an investigation to determine the phagocytosis activity of cells induced after β-Glucans. Our results showed that low-dose Paramylon treatment promoted phagocytosis of MФ against *S. agalactiae*, while high-dose treatment inhibited phagocytosis compared to the control group (**Figure 1D**). On the other hand, Laminarin had no significant effect on phagocytosis at low doses, but a pro-phagocytic effect was observed at high doses (**Figure 1D**). Similar results were obtained in the phagocytosis model of 0.5 (**Supplemental Figure 2A**) and 1.0 μm YG beads (**Supplemental Figure 2B**). Our study revealed that these two β-Glucans triggered distinct immune responses, particularly at different doses. Previous studies have suggested that the high-dose stimulation of β-Glucans can cause apoptosis and thus inhibit the immune responses (21, 30). To test this hypothesis, further verification of apoptosis was performed in the following study.

### 3.2 High-dose Paramylon but not Laminarin leads to MФ apoptosis

**Figure 2:**
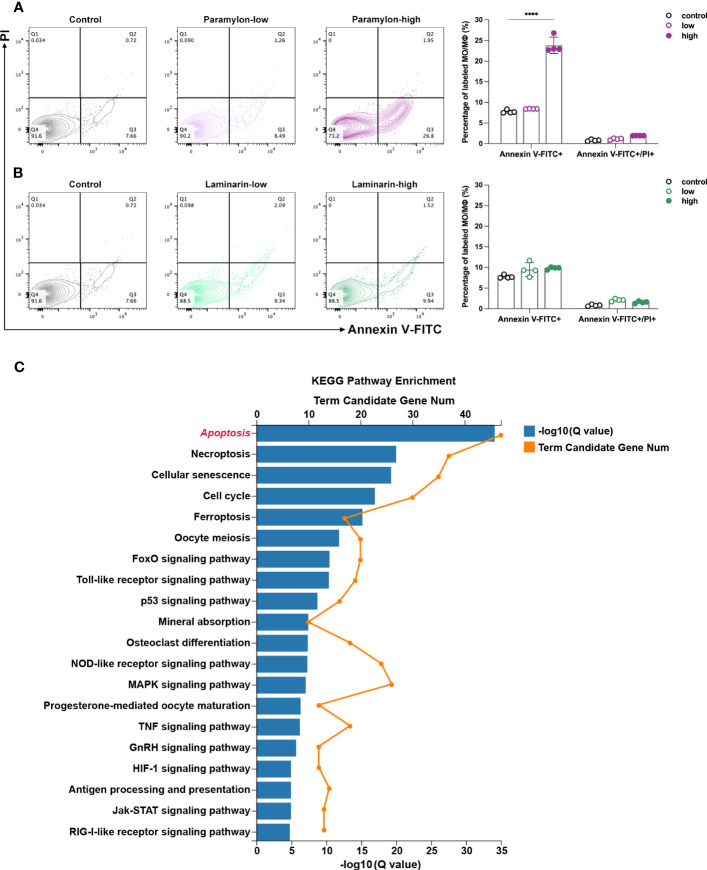
Apoptosis assay and DEGs enrichment. **(A)** Flow cytometry analysis of high-dose Paramylon caused apoptosis in MФ and **(B)** apoptosis was not affected by any dose of Laminarin. All statistical analyses of the data were expressed as means ± SD (n=4). Statistical significance was established by Two-Way ANOVA with Tukey’s multiple comparison tests. * *p* < 0.05, ** *p <*0.01, *** *p* < 0.001, **** *p* < 0.0001. **(C)** KEGG pathway enrichment analysis of 147 up-regulated DEGs in the “Cell growth and death” set in high-dose Paramylon treatment group. Q value < 0.05.

To assess the induction of apoptosis following stimulation with β-Glucans, MФ were treated with both low and high doses of β-Glucans and analyzed via flow cytometry with Annexin V-FITC and PI co-labeling. Our analysis revealed that compared to the control group, the apoptosis of MФ treated with Paramylon increased significantly in a dose-dependent manner, while Laminarin did not induce apoptosis event even at high doses (**Figures 2A, B**). Aside from the visual apoptotic flow assay, the transcriptome data of MФ treated with the high-dose Paramylon also supported the apoptotic results. Using the KEGG pathway classification analysis to profile the up-regulated differentially expressed genes (DEGs), we identified a total of n = 147 DEGs that annotated to “Cell growth and death” (**Supplemental Figure 3**). We further pitched on these 147 DEGs for further KEGG pathway enrichment analysis, and found that the top enriched pathway was “Apoptosis” (**Figure 2C**), providing transcriptional evidence to support the apoptosis caused by the high-dose Paramylon.

These interesting results raised two scientific questions: 1) How does the particulate β-Glucan, Paramylon, triggered apoptosis at high doses? 2) Why dose the solubilized β-Glucan, Laminarin, not induce apoptosis? To identify the underlying mechanism, the following investigations in our study were conducted.

### 3.3 The intrinsic apoptosis pathway is the enforcer of apoptosis in response to high-dose Paramylon stress

**Figure 3:**
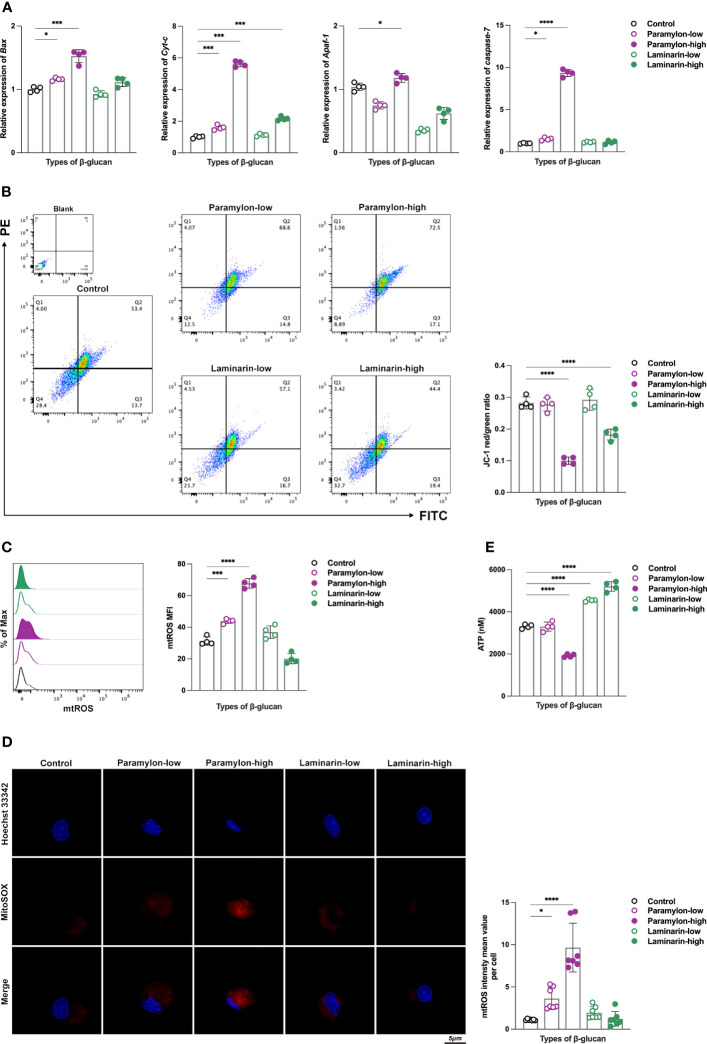
Detection of intrinsic apoptosis-related genes and evaluation of mitochondrial function. **(A)** Relative expression patterns of *Bax*, *Cyt-c*, *Apaf-1* and *caspase-7* mRNA in both low and high-doses β-Glucans treated (12 h) MФ. **(B)** MMP and **(C)** mtROS flow assay. **(D)** mtROS (red) levels of individual cells in different experimental groups. **(E)** ATP production was detected by a multifunctional microplate reader. All statistical analyses of the data were expressed as means ± SD. Statistical significance was established by One-Way ANOVA. * *p* < 0.05, ** *p <*0.01, *** *p* < 0.001, **** *p* < 0.0001. n=7 in **(D)**, and n=4 in the rest.

Relative expression analysis of intrinsic apoptosis-related genes revealed that four representative genes, including *Bax*, a key pro-apoptotic member of the BCL-2 family that regulate intrinsic apoptosis (31), second mitochondria-derived activator of caspases cytochrome c (*Cyt-c*), apoptotic protease-activating factor 1 (*Apaf-1*), and the executioner *caspase-7* (32), were significantly up-regulated after Paramylon challenge, especially at high doses (**Figure 3A**). On the contrary, Laminarin did not exhibit any significant effect on the expression of these genes at any dose, compared to the control group (**Figure 3A**). As impaired mitochondrial function is a dominant factor in the intrinsic apoptotic pathway (33), we evaluated the mitochondrial function of MФ treated with different doses of β-Glucans. We measured mitochondrial membrane potential (MMP), whose decrease is a hallmark event in the early stage of apoptosis (34), and found that high-dose Paramylon and Laminarin caused a forceful loss in MФ MMP (**Figure 3B**). Subsequently, we examined the levels of mitochondrial ROS (mtROS), a byproduct of mitochondrial respiration (35), and found a dose-dependent increase in MФ mtROS resulting from Paramylon stimulation but not Laminarin (**Figure 3C**). These results were in line with the trend in mtROS levels observed for individual cells in the corresponding experimental groups (**Figure 3D**).

As mitochondria are the powerpacks of cells (36), ATP is an indispensable indicator for assessing their function. In this study, we performed an ATP production sensing on MФ treated by varying doses of β-Glucans. Notably, in addition to obtaining the finding of a significant decrease in ATP production in the group treated with high-dose Paramylon (**Figure 3E**), the Laminarin-stimulated group exhibited an unexpected and significant dose-dependent increase in ATP production (**Figure 3E**). These results collectively suggest that high-dose particulate Paramylon, in comparison to solubilized Laminarin, triggers apoptosis by impairing mitochondrial function and up-regulating of genes associated with the intrinsic apoptotic pathway.

### 3.4 Significant upregulation of the metabolite α-Ketoglutarate prevents Laminarin from inducing apoptosis even at high doses

**Figure 4:**
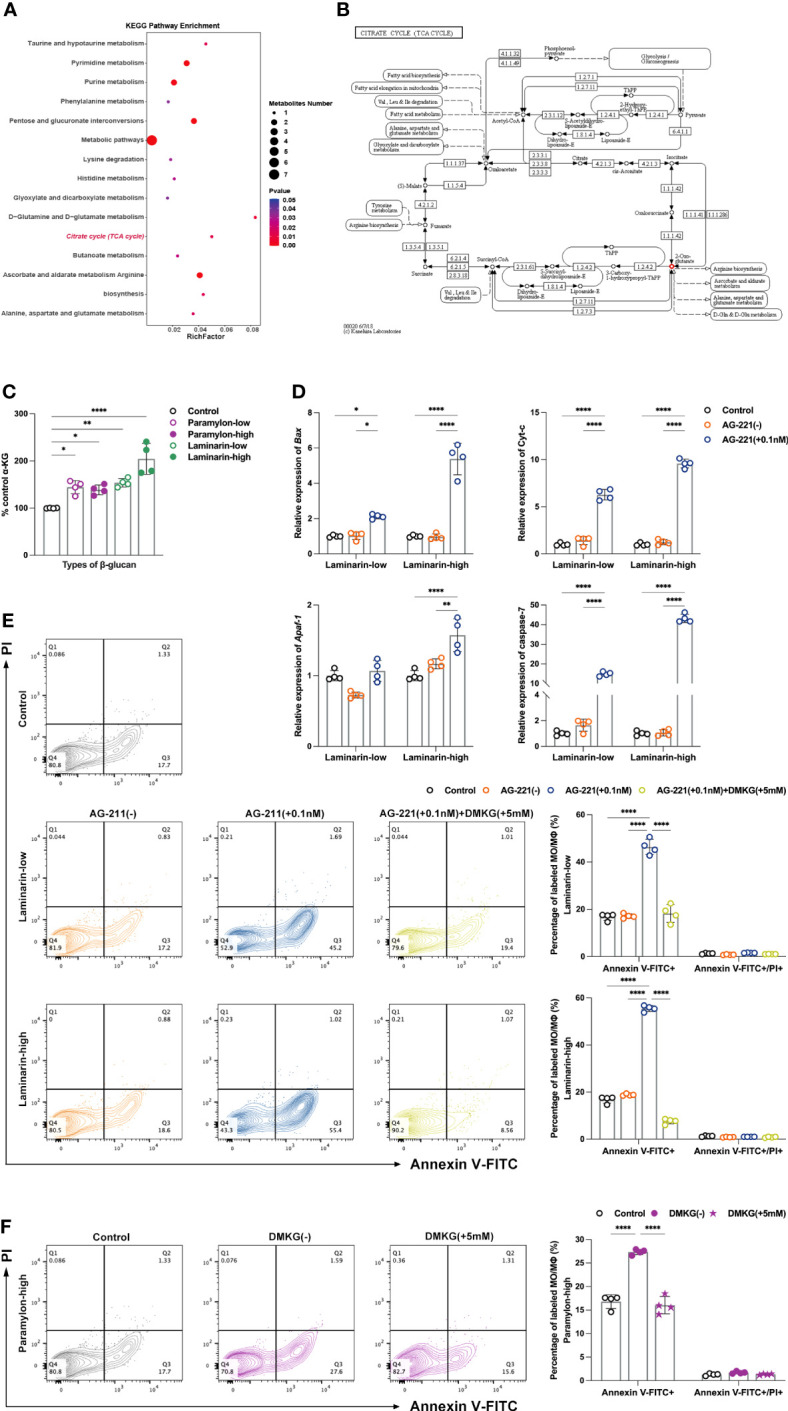
Untargeted metabolomics analysis, α-KG quantification and verification of apoptosis protection. **(A)** Metabolic pathway enrichment analysis of differential metabolites in the low-dose Laminarin treatment group was performed based on the KEGG database. X-axis enrichment factor (RichFactor) is the number of differential metabolites annotated to the pathway divided by all identified metabolites annotated to the pathway. The larger the value, the greater the proportion of differential metabolites annotated to the pathway. The dot size represents the number of differential metabolites annotated to this pathway. P value < 0.05. **(B)** Example diagram of “TCA cycle” enriched by KEGG metabolic pathway. Small box: enzyme, small circle: metabolite (red indicates that the metabolite is a differential metabolite and is up-regulated in the comparison group), arrow: direction of response. 2-Oxo-glutarate=α-KG. **(C)** α-KG quantitative detection, using a multifunctional microplate reader reading at a wavelength of 570 nm. **(D)** Relative expression patterns of *Bax*, *Cyt-c*, *Apaf-1* and *caspase-7* mRNA of MФ treated with Laminarin and AG-221 (0.1 nM). **(E)** Apoptosis assay of MФ treated with Laminarin, or Laminarin and AG-221 (0.1 nM), or Laminarin, AG-221 (0.1 nM) and DMKG (5 mM), and **(F)** apoptosis assay of MФ treated with high-dose Paramylon and DMKG (5 mM). All statistical analyses of the data in **(C-F)** were expressed as means ± SD (n=4). Statistical significance in **(C)** was established by One-Way ANOVA, and in **(D-F)** was established by Two-Way ANOVA with Tukey’s multiple comparison tests. * *p* < 0.05, ** *p <*0.01, *** *p* < 0.001, **** *p* < 0.0001.

The upregulation of ATP in the Laminarin effector group in a dose-dependent manner led us to investigate the metabolomics data of MФ. As the hub of three major nutrient metabolisms, TCA cycle occupies an unquestionably major position in ATP production (37). Surprisingly, KEGG pathway enrichment from the metabolomics data of low-dose Laminarin-stimulated MФ was indeed enriched to the TCA cycle (**Figure 4A**). The only metabolite that was upregulated in the pathway was α-Ketoglutarate (α-KG) (**Figure 4B**), a ketone derivative of glutaric and a key intermediate in the TCA cycle, which is formed by the oxidative decarboxylation of isocitrate by isocitrate dehydrogenase (IDH) (38). Herein, to elucidate the actin of α-KG, we quantified it using a conjugated enzyme assay. Consistent with the hints given by the metabolomics data, the production of α-KG showed a dose-dependent increase after Laminarin treatment compared to the control (**Figure 4C**). We used inhibitor enasidenib (AG-221) (39), to block the conversion of isocitrate to α-KG by IDH2, which mainly exists in mitochondria (40). We found that this inhibition posed an apoptotic risk to MФ, even in an otherwise non-apoptotic Laminarin environment, as evidenced by the largely silent situation of the four intrinsic apoptosis-related genes tested after Laminarin stimulation (**Figure 4D**). To confirm the above results, we added back DMKG, a cell-permeable dimethyl-α-KG (41), to Laminarin-treated MФ with AG-221. Flow cytometry apoptosis assay analysis showed that DMKG backfill rescued apoptosis caused by IDH2 inhibition (**Figure 4E**). Moreover, we also observed that DMKG demonstrated its powerful rescue ability in apoptotic MФ after high-dose Paramylon challenge (**Figure 4F**).

## 4 Discussion

β-Glucans possess immunostimulatory and regulatory properties that are dependent on their specific structures. Structural variations give rise to unique physical and chemical properties, including solubility, molecular size, and viscosity, which impact their biological activities. Comparative studies have revealed differential performances among β-Glucans. Mechanistically, the recognition of β-Glucans is primarily mediated by immune-cell-associated receptors (42). Specifically, Dectin-1, a C-type lectin receptor, has been observed to prefer linear β- (1, 3)-glucans (43), with the minimum β-Glucan unit required for recognition being a 9- or 10-mer of glucose (44, 45). Failure of recognition mechanisms to identify β-Glucans may result in excluded polysaccharides that do not induce a commensurate response. Our evaluation of the immune response phenotypes of MФ showed that Paramylon and Laminarin had completely distinct manifestations. Similar to mammalian studies (12), dose-dependent up-regulation of pro-inflammatory cytokines *IL-1β*, *6*, *8* and *TNF-α* suggested MФ activation, while the effects of Laminarin were much less than that of Paramylon. Laminarin remained relatively silent in terms of ROS and phagocytosis, it was not surprising that the effects of Paramylon were no longer dose-dependent and inhibition occurred at high doses. The reason that Paramylon has shown superior immune response induction compared to Laminarin could relate to its structural features (solubility and molecular weight). Therefore, it is equally important to determine the optimal dose range and chemical structure when employing β-Glucans as feed immunostimulants in aquaculture (46). Soltanian et al. (47) challenged *A. franciscana* with *V. campbellii* to investigate the anti-infectious potential of six commercial β-Glucans. Their findings indicated that the efficacy of β-Glucans in this context depends more on their quality, including factors such as molecular weight, structure ratio of β-1,3/1,6-Glucans, and branching, rather than the quantity administered in the diet. Notably, the presence of specific structural features, such as β-1,3-linkages in the main chain and additional β-1,6 or β-1,4 branch points, was found to be crucial for activating immune cells (48). When considering dosage of β-Glucans in aquaculture, various *in vivo* experimental models of teleost fish have suggested the need for such determinations. Certain studies have indicated that the resistance of aquatic animals to pathogens may not be enhanced, or in some cases, it could even decrease at high concentrations of β-Glucans administration. Therefore, it is crucial for feed manufacturers and fish farmers to carefully consider and regulate the dietary dosage of β-Glucans to avoid any potential negative effects. In terms of MФ response, overstimulation or sustained stimulation may not only result in unmanageable cytokine storms (49), leading to adverse effects such as immunosuppression, but also induce immune tolerance (50), where cells are forced to enter a state of quiescence. This study underscored the significance of considering dose and structural factors when investigating β-Glucans in aquaculture through *in vitro* studies. The observed dose and structure-dependent induction of apoptosis emphasized the importance of monitoring cell death in such studies to mitigate its potential impact on the outcomes.

Soluble β-glucans have been widely used in clinical applications because of their ease of *in vivo* delivery, whereas particulate β-glucans may be more effective in exerting a local immunomodulatory effect (51). This is due to the distinct receptors involved in the activation of immune cells by soluble and particulate β-glucans. Particulate β-glucans activate immune cell directly through the Dectin-1 pathway, whereas soluble β-glucans require complement and complement receptor 3 (CR3)-dependent pathway activation for their antitumor effects (52). Despite the identification of several potential candidates, including members of the C-type lectin family (53) and Toll-like receptor homologs (54), the cellular recognition receptors for β-Glucans have yet to be precisely characterized in teleost fish. Interestingly, we noted cell phagocytosis of Paramylon particles under laser confocal microscopy (results not shown). Paramylon particles were found to be approximately 2-3 μm in size, which falls within the range of pathogenic pathogen sizes. Researchers have stressed the significance of the phagocytosis process in determining immune response of cells to foreign substances and pathogens (55). Beyond the classical pathway of surface receptor recognition and downstream signaling cascades, phagocytosis itself can shape the immune response. This process has been described as “cell tasting, feeling, swallowing and digesting”, in which the ingested object conveys a vast amount of information to the cell, including its physical form, viability, and level of threat. Therefore, due to the unclear nature of β-Glucan receptors in teleost fish remain, an exploration of the immune effects brought by the phagocytosis process of β-Glucans could provide a novel approach to elucidate the immune regulatory mechanism of β-Glucans in teleost fish. Although studies have shown that phagocytosis of Paramylon granules by human lymphocytes does not appear to elicit immune effects (12), the immunoregulatory properties of β-Glucans exhibit significant differences in cell types and species (56, 57). Therefore, investigating the potential immunological implications of β-Glucans granules phagocytosis by teleost MФ still holds significant promise. Furthermore, since cellular phagocytic capacity is not unlimited (58), it is necessary to investigate and validate whether the observed inhibition of phagocytosis within the high-dose Paramylon group in our findings may be linked to a reduction in phagocytic capacity following Paramylon granules ingestion.

Previous *in vitro* and *in vivo* investigations have suggested that β-Glucans can induce apoptosis in teleosts (21, 30), particularly at high doses, which may be interlinked with immunosuppression. Our findings support this association, as high-dose Paramylon treatment resulted in significant apoptosis, whereas Laminarin did not induce apoptosis even at high doses. It is noteworthy that the apoptotic-inducing effects of β-Glucans have been demonstrated to offer potential benefits from multiple perspectives. Numerous studies have highlighted their capacity to stimulate apoptosis in human cancer cells (59), including human melanoma HTB-140 cells (60). The apoptotic effect was characterized by the activation of caspase-3/7, the appearance of phosphatidylserine on the outer surface of the cell membranes, binding of phosphatidylserine to Annexin V-FITC, and a simultaneous decrease in intracellular ATP levels with mitochondrial membrane potential (MMP). These findings underscored the potential of β-Glucans as a promising candidate for anti-tumor therapy. Referring to previous researches on apoptosis, we demonstrated that the intrinsic apoptosis pathway was responsible for the apoptosis induced by high-dose Paramylon. We also noticed the up-regulation of genes involved in the extrinsic apoptotic pathway, such as *Fas* and *FasL*. This result was not unexpected, given the known crosstalk between the intrinsic and extrinsic apoptotic pathways, which ultimately converged at the executioner caspase level (31, 32).

We observed a remarkable increase in ATP production, a metric used to assess mitochondrial function, in a dose-dependent manner following treatment with Laminarin. This suggest that Laminarin may reprogram the metabolic pathways of MФ to reduce cell apoptosis. Consistent with this, our metabolomics data analysis showed an enrichment of the TCA cycle, a central energy metabolic pathway, and identified α-KG as the only significantly differential and up-regulated metabolite in the cycle. Our subsequent quantitative assay results validated the Laminarin dose-dependent increase in α-KG production. Recent researches have demonstrated the immunoprotective properties of the metabolite α-KG, which can activate two demethylases, JMJD3 and PHF8, to prolong the lifespan of nematodes (61). Feeding α-KG to mice has been shown to reduce the levels of systemic inflammatory cytokines, leading to fewer tumors, improved eye health, and longer life expectancy (62). At the cellular level, α-KG is also beneficial for maintaining the pluripotency of embryonic stem cells and plays a mechanistic role in the transcriptional and epigenetic state of stem cells (41). In teleost fish, dietary supplementation of α-KG can enhance the intestinal antioxidant capacity and immune response in carp (*Cyprinus carpio*) infected with *Aeromonas hydrophila* (63). Apart from that, α-KG has been found to exert an anti-apoptotic effect. Nutlin-3a, a small molecule activator of p53, a transcription factor responsive to stress, can be mitigated by α-KG. Upon activation, p53 promotes the expression of downstream target genes, including *P21*, *PUMA*, *Noxa* and *Bax*, which can induce apoptosis (64). The researchers have found that the level of α-KG decreases in cells susceptible to Nutlin-3a-induced apoptosis, while it increases in cells resistant to such apoptosis. Furthermore, the addition of cell-permeable DMKG protects cells from apoptotic response to Nutlin-3a (65). Other existing literature suggests that α-KG has the potential to act as an anti-apoptotic agent by inhibiting the upregulation of *caspase-3* expression (66, 67). Additional experimental models have also suggested that α-KG improves cell viability, enhances mitochondrial respiration, increases TCA cycle efficiency, stabilizes MMP and reduces cytochrome-c release and caspase-3 activation (68). Based on these findings and our results, we hypothesized that Laminarin does not induce apoptosis even at high doses due to the protective effect of α-KG. We tested both forward, using an inhibitor, and reverse, using cell-permeable DMKG, and obtained significant and valid results confirming our hypothesis. At the same time, we further highlighted the potential of α-KG as a rescuer for MФ in an apoptotic environment (high-dose Paramylon treatment). Therefore, as immunometabolism has become a hot topic of research for immunologists, the elucidation of the precise mechanism underlying Laminarin-induced metabolic reprogramming in teleost MФ, as well as the exploration of additional immunomodulatory properties of α-KG, are promising areas for further investigation.

Moreover, a noteworthy study on apoptosis has prompted us to contemplate the intricate nature of this process. A study conducted by Medina (69) revealed that apoptosis is not a mere release of cell contents, but rather apoptotic cells released specific metabolites through defined pathways that regulated the expression patterns of genes associated with immunity and metabolism in surrounding cells. The metabolic mixtures derived from apoptotic cells can induce the expression of anti-inflammatory genes and mitigate inflammatory responses *in vivo*, thereby providing greater immunological significance to apoptotic cells. Accordingly, we speculate whether the cells that underwent apoptosis in response to high-dose Paramylon stimulation in our experiments could also generate more immunomodulatory value via a similar mechanism. Further investigation and experimentation are required to gain more insight into this topic.

**Figure 5:**
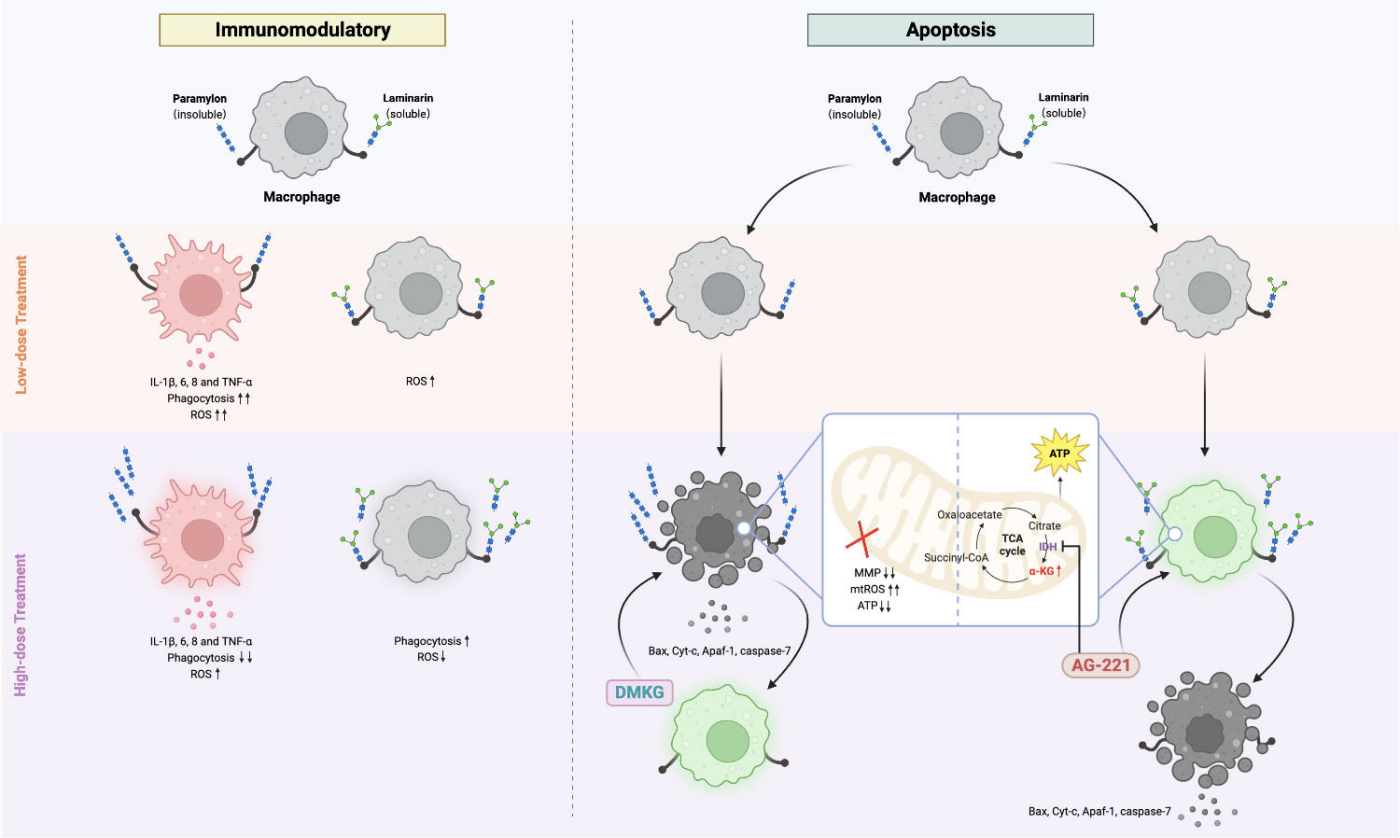
Comparative immunomodulatory and apoptotic effects of Paramylon and Laminarin in teleost macrophages. Paramylon outperforms Laminarin in enhancing teleost macrophage immunomodulatory. High-dose Paramylon triggers apoptosis via mitochondrial apoptotic pathway. Laminarin triggers metabolic reprogramming and upregulates α-ketoglutarate to protect macrophages from apoptosis.

In summary, our study presents the first comparative analysis of the cellular immune responses and apoptotic manifestations induced by Paramylon and Laminarin in teleost fish (**Figure 5**). Our findings highlight the significance of considering the dose and structural properties of β-Glucans when utilizing these polysaccharides as immunomodulatory agents. Furthermore, our discovery and validation of the protective effect of α-KG against apoptosis not only highlight the potential of this compound in immune-protective interventions but also underscore novel insights into how the metabolic switch in immune cells shifts their immune responses.

## Data availability statement

The datasets presented in this study can be found in online repositories. The names of the repositories and accession numbers can be found below: NCBI, PRJNA996550; MetaboLights, MTBLS8254.

## Ethics statement

The animal study was approved by The experimental procedures followed the regulations of the South China Normal University Animal Care and Use Committee (SCNU-SLS-2021-009) and were carried out in accordance with the ARRIVE guidelines. The study was conducted in accordance with the local legislation and institutional requirements.

## Author contributions

ZW performed and analyzed all the experiments with the help from YY. BH and ZW designed the experiments. YY contributed to laser confocal and flow cytometry analysis. JL and XW contributed to real-time PCR and bioinformatics analysis. ZW wrote the original draft. BH and JY revised and edited the final manuscript. PB and ZG contributed to suggestion and critical reading of the manuscript. JY and BH obtained funding. All the authors contributed to the article and approved the submitted manuscript.
