# Supplementary material for: β-Glucans in particulate and solubilized forms elicit varied immunomodulatory and apoptosis effects in teleost macrophages in a dosedependent manner
Source: Front Immunol. 2023 Aug 22;14:1243358. doi: 10.3389/fimmu.2023.1243358 (PMC10477985; doi:10.3389/fimmu.2023.1243358)
Supplement: Supplementary file 1 [file DataSheet_1.docx]

Supplementary Material

β-Glucans in Particulate and Solubilized Forms Elicit Varied Immunomodulatory and Apoptosis Effects in Teleost Macrophages in a Dose-dependent Manner

Zhelin Wu^1^, Yanjian Yang^1^, Jiadong Li^1^, Peter Bossier^3^, Xiayi Wei^1^, Zheng Guo^1^, Biao Han^1*^, Jianmin Ye^1,2*^

*** Correspondence:**Jianmin Ye, Biao Han
jmye@m.scnu.edu.cn, biao.han@scnu.edu.cn

# Supplementary Figures and Tables

**Supplemental Table 1**

Studies on the effects of β-Glucans *in vitro* in teleost fish.

| Species | β-Glucan origin | Dose | Main Effects | Reference | |
| --- | --- | --- | --- | --- | --- |
| Common carp  (*Cyprinus carpio*) | yeast | 100 μg/mL | reactive oxygen and nitrogen radicals increased, *IL-1β*, *IL-6* and *IL-11* were up-regulated. | Pietretti, et al. (70) | |
| Carp  (*Cyprinus carpio*) | yeast | 100 μg/mL | ROS increased. | Vera-Jimenez and Nielsen (71) | |
| Carp  (*Cyprinus carpio*) | yeast | 0 to 1000 μg/mL | a pronounced apoptotic effect on carp pronephric leucocytes with a dose threshold of 500 μg/mL or higher. | | Miest and Hoole (21) |
| Rainbow trout  (*Oncorhynchus mykiss*) | yeast | 6, 30, 60 and 120 μg/ml | *TNFα*, *IL-1β*, *IL-6*, *IL-*8 and *COX2A* were up-regulated after the first stimulation, and *IL-6* was down-regulated after the second stimulation. | Camino Ord´as, et al. (72) | |
| Common carp  (*Cyprinus carpio*) | yeast | 25 μg/mL | members of the C-type lectin family were potential candidates of cellular recognition receptors for β-Glucans in teleost fish. | Petit, et al. (53) | |


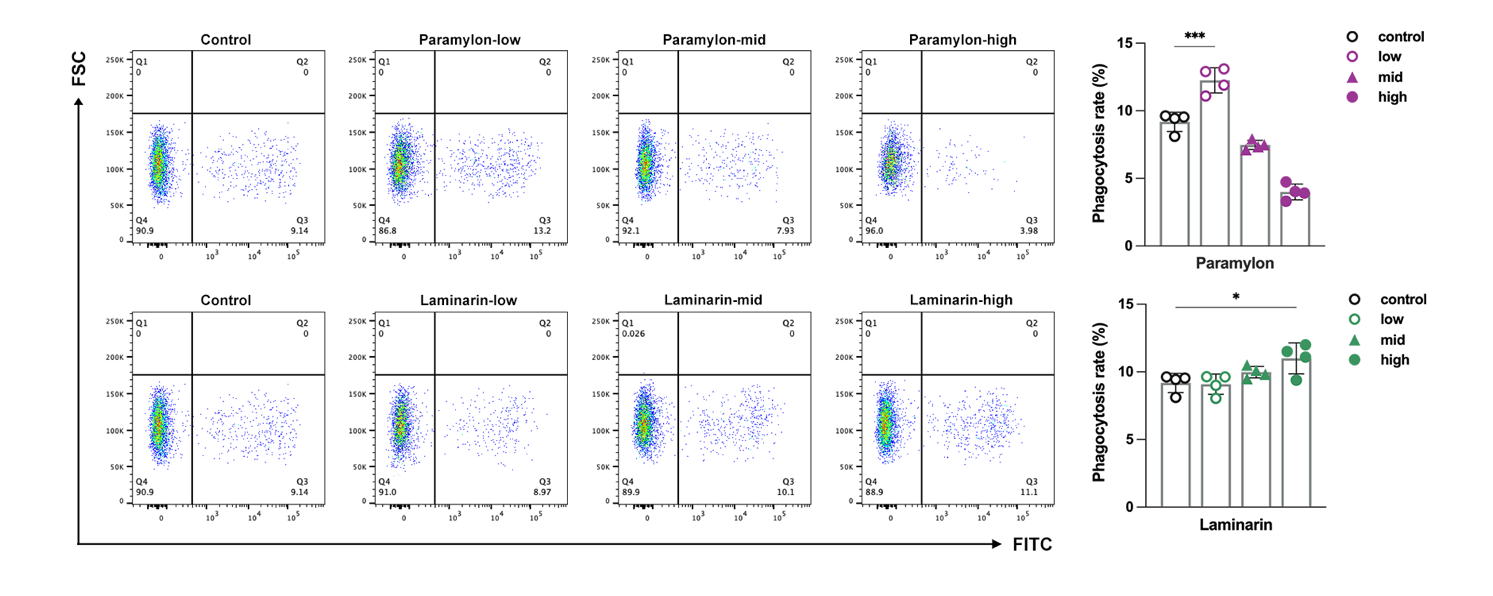
 **Supplemental Figure 1.** Flow phagocytosis assay. Phagocytosis performance of both low, mid and high-dose Paramylon and Laminarin treated (12 h) MФ against *S. agalactiae*. Data were analyzed and presented by FlowJo 10.8.1. All statistical analyses of the data were expressed as means ± SD (n=4). Statistical significance was established by One-Way ANOVA. * *p* < 0.05, ** *p* <0.01, *** *p* < 0.001, **** *p* < 0.0001.


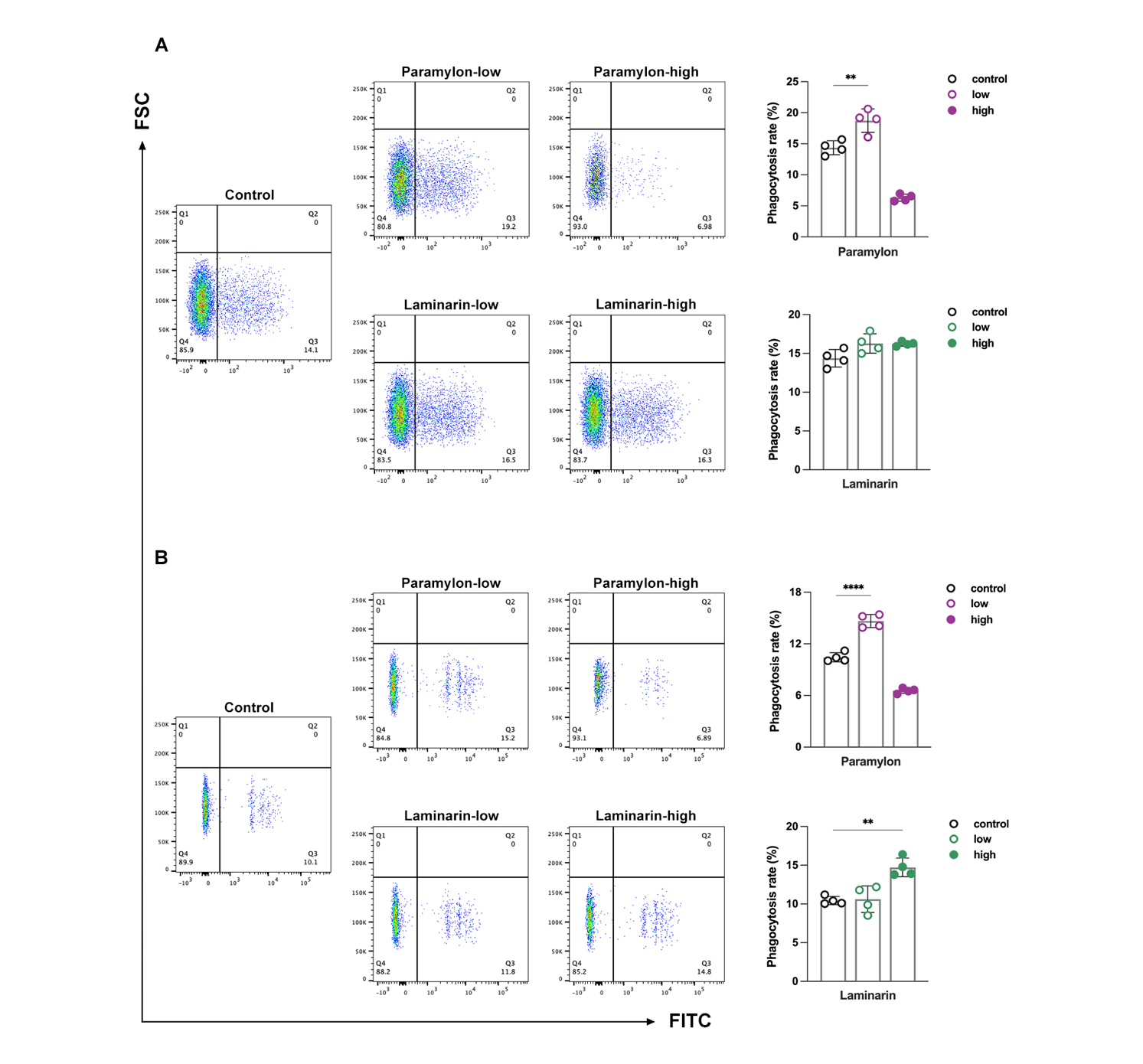


**Supplemental Figure 2.** Flow phagocytosis assay. (A) The phagocytosis model of 0.5 and (B) 1.0 μm YG beads were built to evaluate phagocytosis performance of MФ treated with β-Glucans (12h) at both low and high doses. Data were analyzed and presented by FlowJo 10.8.1. All statistical analyses of the data were expressed as means ± SD (n=4). Statistical significance was established by One-Way ANOVA. * *p* < 0.05, ** *p* <0.01, *** *p* < 0.001, **** *p* < 0.0001.


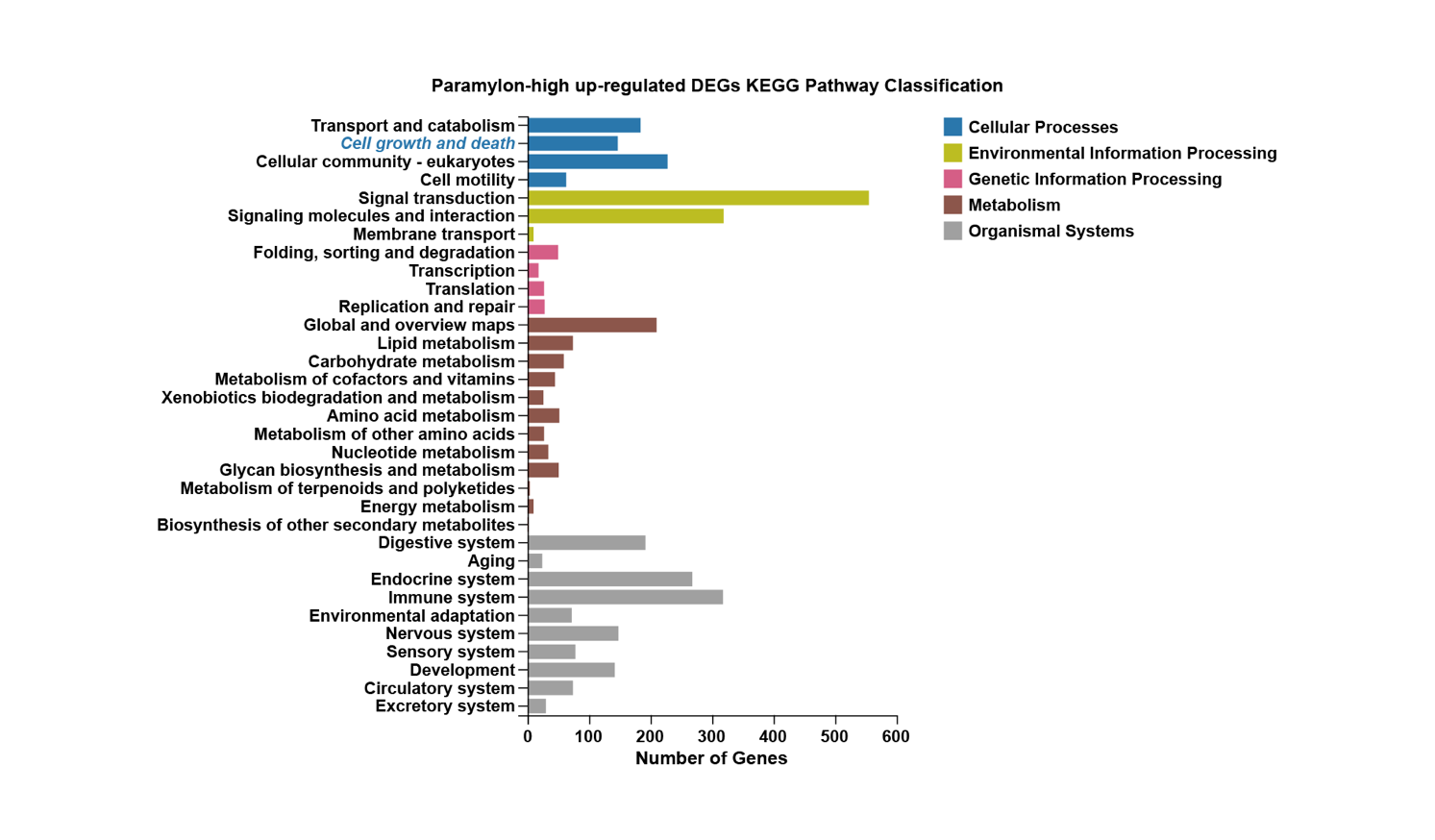


**Supplemental Figure 3.** KEGG pathway classification analysis of up-regulated DEGs in high-dose Paramylon treatment group, a total of n=147 DEGs were annotated into “Cell growth and death”. log2FC > 1, Q value < 0.05.

**References**

70. Pietretti D, Vera-Jimenez NI, Hoole D, Wiegertjes GF. Oxidative burst and nitric oxide responses in carp macrophages induced by zymosan, MacroGard® and selective dectin-1 agonists suggest recognition by multiple pattern recognition receptors. *Fish Shellfish immunol* (2013) 35:847-857. doi: 10.1016/j.fsi.2013.06.022

71. Vera-Jimenez NI, Nielsen ME. Carp head kidney leukocytes display different patterns of oxygen radical production after stimulation with PAMPs and DAMPs. *Mol Immunol* (2013) 55:231-236. doi: 10.1016/j.molimm.2013.01.016

72. Ord´as MC, Gonz´alez-Torres L, Arense P, Heavyside R, Zarza C, Tafalla C. Analysis of immunostimulatory responses and immune tolerance to β-glucans in rainbow trout cell lines. *Aquaculture* (2021) 541:736805. doi: 10.1016/j.aquaculture.2021.736805
